# Impact of Systemic Therapy on Fertility in Women with Early-Stage Breast Cancer

**DOI:** 10.1007/s12609-023-00516-z

**Published:** 2024-01-03

**Authors:** Kelsey H. Natsuhara, A. Jo Chien

**Affiliations:** 1 Division of Hematology & Oncology, Department of Medicine, University of California San Francisco, 1825 4th St, San Francisco, CA 94158, USA

**Keywords:** Breast cancer, Fertility, Fertility preservation, Reproductive health

## Abstract

**Purpose of Review:**

Fertility concerns are common among young women diagnosed with breast cancer, as systemic therapy increases the risk of premature ovarian insufficiency and delays family planning. Here, we review the impact of systemic therapies, including chemotherapy, endocrine therapy, HER-2 directed therapy, PARP inhibitors, and immunotherapy, on ovarian reserve.

**Recent Findings:**

With an improved understanding of disease biology, fewer women are treated with gonadotoxic chemotherapy. There are limited data on the fertility impact of novel targeted treatments and immunotherapy, though preclinical and preliminary studies suggest an impact on fertility is possible. Notably, a recent study investigated the outcomes in women who interrupted adjuvant endocrine therapy to attempt pregnancy.

**Summary:**

Further research is needed to characterize the fertility impact of novel therapies in breast cancer. Individualized fertility counseling should be offered to all women to discuss the possible impact of therapy on ovarian reserve and options for fertility preservation and timing of pregnancy.

## Introduction

The incidence of breast cancer is rising, with an estimated 12,000 new cases each year in the USA [1]. Furthermore, breast cancer is one of the most common malignancies among women under the age of 40 years, with women 15–39 years old making up 18% of all new breast cancer diagnoses in the USA [2]. Fertility concerns are critically important to younger patients and affect quality of life and treatment decision-making [3, 4]. Not only do young women with a new breast cancer diagnosis face the risk of premature ovarian insufficiency due to the impact of systemic therapies, but they also must typically delay family planning due to treatment. Approximately two-thirds of young women with breast cancer have hormone receptor (HR)-positive disease and will face an additional 5–10 years of adjuvant endocrine therapy, which can further delay fertility plans [5]. Fertility naturally decreases with age, and while a fertile 30-year-old woman has approximately a 20% chance of pregnancy per cycle, a 40-year-old woman has less than a 5% chance of pregnancy per cycle [6].

These, among other factors, can affect young breast cancer patient’s treatment decisions. In one study of 620 women diagnosed with breast cancer under the age of 40 years, 26% reported that concerns about fertility affected their treatment decisions, including such decisions as declining chemotherapy, choosing one regimen over another, declining endocrine therapy, or discontinuing endocrine therapy before the recommended 5 years [3]. However, when women receive specialized counseling about fertility and fertility preservation, studies have shown decreased regret and improved quality of life [7]. Thus, it is of paramount importance that women have access to accurate information and resources to make informed treatment about their fertility options. Here, we summarize the current body of research on the impact of systemic therapies for early-stage breast cancer on fertility for young women.

### Impact of Cytotoxic Chemotherapy

Cytotoxic chemotherapy has long been the mainstay for systemic breast cancer treatment, but also carries a high risk of premature ovarian insufficiency [8]. Studies into the impact of chemotherapy on fertility rates have largely focused on amenorrhea and surrogate markers of fertility, such as anti-Müllerian hormone (AMH) levels [9, 10]. The resumption of menses has been used as a proxy for fertility, with most women resuming menses in the first 6–9 months after treatment [11]; however, it is well understood that the return of menses is not a surrogate for fertility and more reliable surrogates are needed. AMH is a sensitive indicator of ovarian follicular reserve and is used as a fertility surrogate. While AMH consistently falls during chemotherapy with slow recovery after the end of treatment, though typically not to pre-treatment levels, increasing AMH levels are not always predictive of fertility [10]. For example, in an observational study of women with breast cancer who received chemotherapy and conceived without reproductive assistance, there was no association found between baseline and end of treatment AMH levels and occurrence of pregnancy [12]. In order to better understand the true impact on fertility and pregnancy after chemotherapy, more reliable and validated surrogate markers are needed.

Despite the limitations in accurately estimating fertility, it is important to counsel women on the rates of amenorrhea and impact of chemotherapy on fertility prior to treatment. Older regimens, like cyclophosphamide, methotrexate, and 5-fluorouracil (CMF), demonstrated rates of amenorrhea around 40% for women ≤ 40 years [13]. Modern day chemotherapy regimens for patients with early-stage breast cancer include anthracycline, cyclophosphamide, and a taxane (ACT) or a taxane plus cyclophosphamide (TC). Rates of amenorrhea for women ≤ 40 years receiving ACT are estimated to range from 13 to 60% and for TC are estimated around 7% [14–16]. Some women with HER2+ disease may receive a taxane and a platinum, such as carboplatin, and this regimen is thought to have less ovarian toxicity than ACT, though the rates of treatment-related amenorrhea are poorly characterized [17, 18]. Similarly, there is a paucity of data on the fertility impact of capecitabine, which has an overall survival benefit for patients with triple negative breast cancer and residual disease after neoadjuvant therapy [19]. This is particularly significant, since triple negative breast cancer often impacts younger women and the additional 6 months of treatment with capecitabine is likely to impact both fertility and family planning timelines.

Notably, with the advent of multi-gene assays like Oncotype DX and MammaPrint, fewer patients are receiving cytotoxic chemotherapy as we can better predict which patients will benefit most based on their disease biology [20, 21]. However, based on prospective randomized studies, chemotherapy benefit does appear to differ based on age, with greater benefit seen in younger women [22, 23]; thus, these molecular tests may not spare young women from chemotherapy as much as older postmenopausal women. It is possible that for some premenopausal women, chemotherapy benefit is at least in part due to the ovarian suppression induced by chemotherapy, and not necessarily from chemotherapy itself. This is currently an important outstanding question being investigated in a prospective randomized trial (NCT05879926); however, both mechanisms significantly impact future fertility and highlight the need for ongoing efforts to improve fertility preservation.

### Fertility Preservation and Safety

The American Society of Clinical Oncology recommends that young women with breast cancer be referred early to a reproductive endocrinology and infertility specialist to discuss their options [24]. One area of interest is the use of LHRH analogues for ovarian function suppression (OFS) during chemotherapy to preserve ovarian function. However, studies have shown mixed results. While multiple studies have clearly demonstrated an increased rate of menstrual return with the use of LHRH analogues for OFS during chemotherapy, as stated previously, studies have not shown a consistent increase in rates of pregnancy [25–27]. Currently, oocyte and embryo cryopreservation are the best established and most successful methods of fertility preservation (FP) for women [28•]. Women should be appropriately informed of their options for FP and understand that, while we do not have prospective data, there are multiple retrospective studies that have demonstrated that FP is not associated with worse breast cancer outcomes or treatment delays and is generally considered safe and effective [28•, 29•].

In a retrospective review of 349 women ≤ 45 years with a new diagnosis of stages I–III breast cancer at Memorial Sloan Kettering by Crown et al., overall survival (OS) and recurrence-free survival (RFS) rates were similar between women who underwent FP and those who did not [28•]. Both HR+ and HR− patients were included, and rates of HR positivity were similar between groups. The 5-year OS and RFS rates for women who underwent FP versus those who did not were similar (OS 98.2% vs 95.9%; RFS 92.1% vs 89.7%). Furthermore, this study found that FP was not associated with breast cancer treatment delays. For patients receiving adjuvant chemotherapy (85.5% of patients), the median interval was 7 weeks between surgery and chemotherapy, both for women who underwent FP and those who declined. Similar findings were reported in a cohort of 153 women ≤ 40 years with newly diagnosed early breast cancer, where 71 (46.4%) chose to undergo FP. Authors Wang and Tesch et al. found no difference in 4-year invasive breast cancer free survival (iBCFS, HR 1.006, 95% CI 0.416–2.438) or OS (HR 0.789, 95% CI 0.210–2.956) between those who underwent FP and those who did not [29•]. The median time from diagnosis to initiation of ovarian stimulation was 55 days (range 9–138 days). Furthermore, while it was noted that patients undergoing FP were more likely to be ECOG 0, there was no difference with regard to baseline tumor stage, HR status, and age at diagnosis between those that underwent FP and those that did not, suggesting that baseline clinical risk was balanced between the groups [29•]. In the study by Crown et al., women who declined FP were more likely to be older (median 37 vs 33 years) and, similarly, there were no differences in baseline tumor stage or HR status [28•]. Overall, these studies support that FP is not associated with worse outcomes or delays in treatment and should be presented to patients as an option.

The majority of the safety data related to FP in early-stage breast cancer comes from patients treated in the adjuvant setting, after the tumor has been removed. In the last decade, the use of NACT has increased, particularly in young patients who often present with higher rates of triple negative cancer and biologically chemo-sensitive tumors where NACT is indicated. Some studies have shown that patients who choose FP are significantly more likely to receive NACT vs. those who declined FP [29•], while other studies have shown that receipt of NACT is a negative predictor of FP [30]. There are additional considerations associated with FP in the neoadjuvant setting. Egg retrieval requires ovarian stimulation, which can cause transiently increased estradiol levels, and this can generate concerns about oncologic outcomes in the setting of a hormonally driven cancer [30]. In order to address this, studies have shown that using tamoxifen or letrozole during ovarian stimulation reduces peak estradiol levels during FP, and this has become a standard part of many FP protocols [31].

Furthermore, the perceived delays associated with FP are often a deterrent for women and their providers. Current “random start” FP protocols, which do not wait for menses to begin, can often be completed during a 2–3 week period, which can coincide with the time when patients are awaiting surgery or chemotherapy scheduling, to prevent treatment delays [29•]. In a retrospective study of women enrolled in the ISPY-2 neoadjuvant chemotherapy trial, there was no differences in time to NACT start between women who pursued FP and those who did not (39.8 vs 40.9 days, respectively) [32]. To our knowledge, there are no published prospective studies evaluating FP and oncologic outcomes specifically in breast cancer patients receiving NACT. However, retrospective studies with subgroup analyses of patients who received NACT do not show differences in recurrence rates [33]. Notably, in the aforementioned study by Wang et al., rates of NACT were 93% among women undergoing FP versus 67% among who declined FP [29•]. Thus, given the high rates of NACT in this study and similar rates of recurrence rates and OS, this lends support to the safety of ovarian stimulation in the neoadjuvant setting. However, additional studies with longer follow-up, detailed patient and tumor characteristics, and modern stimulation protocols are needed. Overall, these studies provide evidence that FP protocols using ovarian stimulation are not associated with higher rates of breast cancer recurrence or significant treatment delays and all women should be appropriately counseled on their options for FP prior to treatment initiation.

### Impact of Endocrine Therapy and Interruptions for Pregnancy

As previously stated, one of the challenges of fertility in breast cancer is not only the impact of cytotoxic chemotherapy on ovarian reserve and delays in family planning associated with initial treatment, but also the additional 5–10 years of endocrine therapy recommended for women with HR+ breast cancer. In one study of pre-menopausal women who received chemotherapy and went on to receive tamoxifen, women on tamoxifen had higher rates of amenorrhea 1–2 years after chemotherapy receipt; however, by 3 years, there was no difference in return of menses, suggesting the ovarian suppression from tamoxifen is temporary and reversible [34]. Furthermore, other studies have shown that tamoxifen use in the absence of chemotherapy did not impact the mean age of menopause onset, suggesting that tamoxifen alone is unlikely to significantly accelerate ovarian aging [35].

However, for women who would like to become pregnant before completing endocrine therapy, an open question in the field has been the impact of interrupting endocrine therapy to allow patients to attempt pregnancy and breastfeed. The POSITIVE (Pregnancy Outcome and Safety of Interrupting Therapy for Women with Endocrine Responsive Breast Cancer) trial, by Partridge et al., sought to answer this question [36••]. In this trial, 516 women ≤ 42 years old with stages I–III HR+ breast cancer who had received 18–30 months of adjuvant endocrine therapy interrupted therapy to attempt pregnancy. Patients could interrupt endocrine therapy for up to 2 years to allow for attempting pregnancy, delivery, and breastfeeding if desired. After pregnancy and/or breast feeding were completed or after unsuccessful conception, endocrine therapy was resumed for the planned 5–10 years of therapy. The number of breast cancer events was close to, but did not exceed, the primary analysis safety threshold, and there was no statistically significant difference in the 3-year incidence of breast cancer events compared to an external control cohort from the SOFT/TEXT trial (8.9% vs 9.2%, respectively). Thus, the authors concluded from this data that temporary interruption of endocrine therapy to attempt pregnancy was not associated with a greater short-term risk of breast events, though longer follow-up is needed.

Notably, in this study, 93.4% of patients had stage I or II disease, so it is possible that these findings are biased by the “healthy mother” effect. Meaning that women who had lower risk disease or who were overall healthier were more likely to feel comfortable interrupting endocrine therapy to attempt pregnancy than patients who had higher risk disease. Another concern during the design of the trial was that patients would not resume endocrine therapy. However, they found that most patients resumed therapy within the recommended timeframe, with only 15.4% of patients not resuming, which was similar to the rates of endocrine therapy discontinuation, approximately 20%, in the SOFT and TEXT trials [37]. Lastly, the authors note that they will need to await the 10-year follow-up data to fully understand the long-term risk of interruptions in endocrine therapy. The POSITIVE trial provides important data for discussions about the risks and benefits of endocrine therapy interruption to attempt pregnancy; however, these results must always be used in the context of the patient’s individual clinical risk and personal circumstances. These data may help some women who otherwise may have declined endocrine therapy all together, as seen in previous studies [3], feel more comfortable starting treatment, knowing they can take a break from therapy in the future if they desire pregnancy.

### Impact of HER-2 Directed Therapy

Data on the impact of HER-2 directed therapy on fertility are limited. In the NRG Oncology/NSABP B-47 menstrual history study, the authors evaluated the impact of chemotherapy with or without trastuzumab on treatment-related amenorrhea in over 1400 pre- and perimenopausal women [38•]. They found that trastuzumab did not contribute to higher rates of amenorrhea, with 84% of patients in the trastuzumab group who were amenorrheic vs 86.3% in the non- trastuzumab group at 12 months. This finding is similar to that of previous studies, such as the ALTTO trial, which evaluated the impact of adjuvant lapatinib with or without trastuzumab, where the authors also found no difference in treatment-related amenorrhea rates (approximately 72–74% in all groups) [15].

With the advent of HER2-directed antibody drug conjugates (ADCs), like ado-trastuzumab-emtansine (T-DMI) and trastuzumab-deruxtecan (T-DXd), more research is needed into the impact of this drug class on fertility. In the ATEMPT trial, patients with T1N0 HER2+ early-stage breast cancer were randomized to T-DMI or paclitaxel + trastuzumab (TH), and rates of treatment-related amenorrhea among pre-menopausal women at 18 months were 24% after T-DM1 and 50% after TH (*p*=0.045) [39•]. While the amenorrhea rates were lower in the T-DM1 group at the 18-month timepoint, notably, the amenorrhea rate seemed to increase in the T-DM1 arm over time, likely because patients in the T-DM1 arm received cytotoxic treatment for 12 months whereas in the TH arm, cytotoxic therapy was administered only in the first 12 weeks of treatment. This study provides key information in characterizing the fertility impact of ADCs, which are becoming a cornerstone of breast cancer treatment. To our knowledge, there are no published studies on rates of treatment-related amenorrhea or the impact of fertility from T-DXd, which is now being investigated in the post-neoadjuvant setting in patients with early breast cancer. However, as this drug becomes more widespread in the breast cancer treatment landscape, understanding its impact for young women on fertility will be critical.

### Impact of Immunotherapy

Immunotherapy has dramatically changed the landscape of cancer care. The checkpoint inhibitor (CPI) pembrolizumab is approved for the treatment of early-stage triple negative breast cancer based on results from the neoadjuvant KEYNOTE 522 study [40] which is particularly relevant for young women given the higher rates of triple negative disease among young women. However, the impact of CPIs on fertility and pregnancy has yet to be clearly defined. Data largely comes from pre-clinical studies, case reports, and aggregated adverse event data from clinical trials. The risk of adverse fetal outcomes is thought to be related to the maternal immune response to the fetus rather than direct cytotoxic effects, and as such, the National Comprehensive Cancer Network advises all patients of reproductive age to use effective contraception during and for at least 5 months after the last dose of immunotherapy [41].

Immune-related endocrinopathies can impact fertility and pregnancy. There are reports of CPI-induced primary hypogonadism, though this appears to be a rare side effect and the evidence base comes from care reports and case series [42]. More commonly, secondary hypogonadism arises from hypophysitis and hypothyroidism, which are well-known immune-related adverse events [42, 43]. The estimated rates of hypophysitis are 0.5–1% for anti-PD1 therapy, 5–6% for anti-CTLA4 therapy, and 9–10% for combination therapy [44]. Disruption of the anterior pituitary gland leads to impaired secretion of follicle-stimulating hormone and luteinizing hormone, which can lead to ovulatory dysfunction or premature menopause [45, 46]. Hypothyroidism, which are commonly seen with pembrolizumab and other anti-PD1 therapies, can also lead to reduced fertility. Inadequately treated hypothyroidism is associated with increased maternal risks, including hypertension, placental abruption, pre-term birth, and low birth weight [47, 48].

There are few studies of the impact of CPIs on fertility in humans. Current published studies only include patients with melanoma and are limited by small sample sizes. In one small study of 28 women under 35 years with melanoma receiving ipilimumab, AMH levels were measured before and after treatment and were found to significantly decrease after CPI therapy [49]. In another small study of women under the age of 40 years with stage III–IV melanoma, median AMH levels and antral follicle counts determined by pelvic ultrasound were lower in the group who were treated with CPIs (*n*=6) vs those who did not (*n*=6) [50]. These data suggest that CPIs may impact ovarian reserve and possibly female fertility directly and larger studies are needed to validate these findings. As the role of immunotherapy continues to expand in all tumor types, it will be of increasing importance to study the direct and indirect impact of CPI and other immunotherapies on fertility in our younger population.

### Impact of PARP Inhibitors

There is also a paucity of data regarding the impact of PARP inhibitors on fertility. Olaparib, a PARP inhibitor, is approved for patients with germline BRCA mutations, based on data from the OlympiA trial that showed an OS benefit in patients with high-risk BRCA-associated early breast cancer who took 1 year of adjuvant olaparib [51]. Patients with germline BRCA mutations often develop breast cancer at a younger age, and thus are more likely to present in their reproductive years [52]. Some studies have shown an association between germline BRCA mutations and decreased ovarian reserve, as BRCA genes are essential to oocyte survival in preventing potential genetic stress [53, 54]. Similarly, the PARP proteins are involved in double-strand break repair and use of olaparib in pre-clinical rodent models was associated with a decrease in ovarian reserve [55]. Thus, it is critical to better understand the impact of olaparib and other PARP inhibitors on fertility, given patients with BRCA mutations are already at increased risk of infertility due to decreased ovarian reserve, and may be recommended to complete up to a year of treatment with olaparib, which may have additional impact on fertility.

## Conclusion and Future Directions

Breast cancer is one of the most common malignancies in young women and the impact of systemic therapy on fertility is a critical concern for patients. Studies have shown that young patients experience significant distress about fertility loss, but this can be mitigated with appropriate counseling on risks of treatment and FP options [3, 7]. Furthermore, multiple studies have now been published that demonstrate no increased risk of recurrence or significant delays in treatment when women undergo FP before or during cancer treatment, in both the adjuvant and neoadjuvant settings [28•, 29•], which may help young women feel more comfortable pursuing FP if they desire. Additionally, large ongoing registry studies, like the PREgnancy and FERtility (PREFER) study (NCT02895165), which is enrolling 1000 premenopausal patients with newly diagnosed breast cancer and following them for up to 15 years after FP, will be helpful in addressing some of the field’s outstanding questions about the safety and efficacy of FP [56].

As the field moves toward better characterization of tumor biology and more individualized decision-making regarding (neo)adjuvant systemic therapy, the hope is that we will spare more young patients from unnecessary chemotherapy, and provide them with less gonadotoxic de-escalated options, thereby improving fertility and reproductive options. However, for many patients, treatment will need to be more intensive and longer, including the addition of targeted therapy and immunotherapy to chemotherapy, as well as the addition of 1–2 years of additional adjuvant targeted therapy in higher risk patients. It is imperative that we study the impact of each of these novel targeted therapies on fertility and pregnancy outcomes. In order to do so, research using more reliable markers of ovarian reserve than menstrual status are needed, as is research looking at the long-term impacts on pregnancy outcomes. One such study is the MotHER trial, a prospective study evaluating women exposed to trastuzumab and/or pertuzumab during pregnancy or within 6 months prior to conception [57]. Generating high-quality data on the fertility and reproductive impact of novel treatments including immunotherapy, ADCs, and small molecule inhibitors should be prioritized in order to help patients make informed decisions related to fertility preservation, family building, and breast cancer treatment.

## Data Availability

No datasets were generated or analyzed for this manuscript.
